# Structures of TOG1 and TOG2 from the human microtubule dynamics regulator CLASP1

**DOI:** 10.1371/journal.pone.0219823

**Published:** 2019-07-19

**Authors:** Jonathan B. Leano, Kevin C. Slep

**Affiliations:** 1 Department of Biochemistry & Biophysics, University of North Carolina, Chapel Hill, North Carolina, United States of America; 2 Program in Molecular and Cellular Biophysics, University of North Carolina, Chapel Hill, North Carolina, United States of America; 3 Department of Biology, University of North Carolina, Chapel Hill, North Carolina, United States of America; Virginia Commonwealth University, UNITED STATES

## Abstract

Tubulin-binding TOG domains are found arrayed in a number of proteins that regulate microtubule dynamics. While much is known about the structure and function of TOG domains from the XMAP215 microtubule polymerase family, less in known about the TOG domain array found in animal CLASP family members. The animal CLASP TOG array promotes microtubule pause, potentiates rescue, and limits catastrophe. How structurally distinct the TOG domains of animal CLASP are from one another, from XMAP215 family TOG domains, and whether a specific order of structurally distinct TOG domains in the TOG array is conserved across animal CLASP family members is poorly understood. We present the x-ray crystal structures of *Homo sapiens* (*H*.*s*.) CLASP1 TOG1 and TOG2. The structures of *H*.*s*. CLASP1 TOG1 and TOG2 are distinct from each other and from the previously determined structure of *Mus musculus* (*M*.*m*.) CLASP2 TOG3. Comparative analyses of CLASP family TOG domain structures determined to date across species and paralogs supports a conserved CLASP TOG array paradigm in which structurally distinct TOG domains are arrayed in a specific order. *H*.*s*. CLASP1 TOG1 bears structural similarity to the free-tubulin binding TOG domains of the XMAP215 family but lacks many of the key tubulin-binding determinants found in XMAP215 family TOG domains. This aligns with studies that report that animal CLASP family TOG1 domains cannot bind free tubulin or microtubules. In contrast, animal CLASP family TOG2 and TOG3 domains have reported microtubule-binding activity but are structurally distinct from the free-tubulin binding TOG domains of the XMAP215 family. *H*.*s*. CLASP1 TOG2 has a convex architecture, predicted to engage a hyper-curved tubulin state that may underlie its ability to limit microtubule catastrophe and promote rescue. *M*.*m*. CLASP2 TOG3 has unique structural elements in the C-terminal half of its α-solenoid domain that our modeling studies implicate in binding to laterally-associated tubulin subunits in the microtubule lattice in a mode similar to, yet distinct from those predicted for the XMAP215 family TOG4 domain. The potential ability of the animal CLASP family TOG3 domain to engage lateral tubulin subunits may underlie the microtubule rescue activity ascribed to the domain. These findings highlight the structural diversity of TOG domains within the CLASP family TOG array and provide a molecular foundation for understanding CLASP-dependent effects on microtubule dynamics.

## Introduction

Microtubules are highly dynamic, polarized eukaryotic cellular polymers [1–4]. Microtubules are composed of αβ-tubulin heterodimers that polymerize through lateral and longitudinal associations to form a cylindrical, polarized lattice with α-tubulin and β-tubulin exposed at the microtubule minus and plus end respectively. Microtubule dynamics occur at both the plus and minus ends, but are primarily focused at the plus end. During phases of polymerization, tubulin heterodimers with GTP bound at the exchangeable site on β-tubulin incorporate into the lattice and define the “GTP cap” [5–7]. Once incorporated into the lattice, the GTP in the exchangeable site is hydrolyzed to GDP. It is the structural transition of tubulin subunits in the microtubule lattice from a GTP-bound state to a GDP-bound state that underlies the polymer’s dynamic instability. Collectively, dynamic instability includes phases of polymerization, depolymerization, and pause, with the transition to depolymerization termed catastrophe, and the transition out of depolymerization termed rescue. While dynamic instability is inherent to microtubules it is highly regulated in space and time by a host of microtubule associated proteins (MAPs). A key subset of MAPs include microtubule plus end binding proteins that localize to polymerizing microtubule plus ends [8–11].

Many microtubule plus end binding proteins form a complex network of interactions both with each other and the microtubule polymer. A master plus end binding protein family is the end binding (EB) protein family (*e*.*g*. *H*.*s*. EB1, EB2, and EB3) that preferentially binds the post-hydrolysis GDP^.^P_i_ microtubule state, best-mimicked by GTPγS-bound microtubules [12]. EB members use a dimerization domain to recruit SxIP or LxxPTPh motif-containing proteins to the microtubule plus end [10,13–15]. Two prime animal MAP families that bind tubulin and are recruited to microtubule plus ends by EB proteins (either directly or indirectly) are the Cytoplasmic Linker-Associated Protein (CLASP) family and the XMAP215 family of microtubule polymerases [16–20]. Both CLASP and XMAP215 members are critical for proper interphase microtubule dynamics as well as mitotic spindle structure and dynamics [16,21–23]. While XMAP215 family members promote microtubule polymerization, CLASP family members promote microtubule pause, potentiate rescue, and limit catastrophe [16,24–29]. Mutations in CLASP family members result in aberrant microtubule dynamics that manifest in phenotypes ranging from abnormal mitotic spindle structure to defects in axon guidance [23,30–32]. How CLASP and XMAP215 family members mechanistically regulate microtubule dynamics is poorly understood.

**Fig 1:**
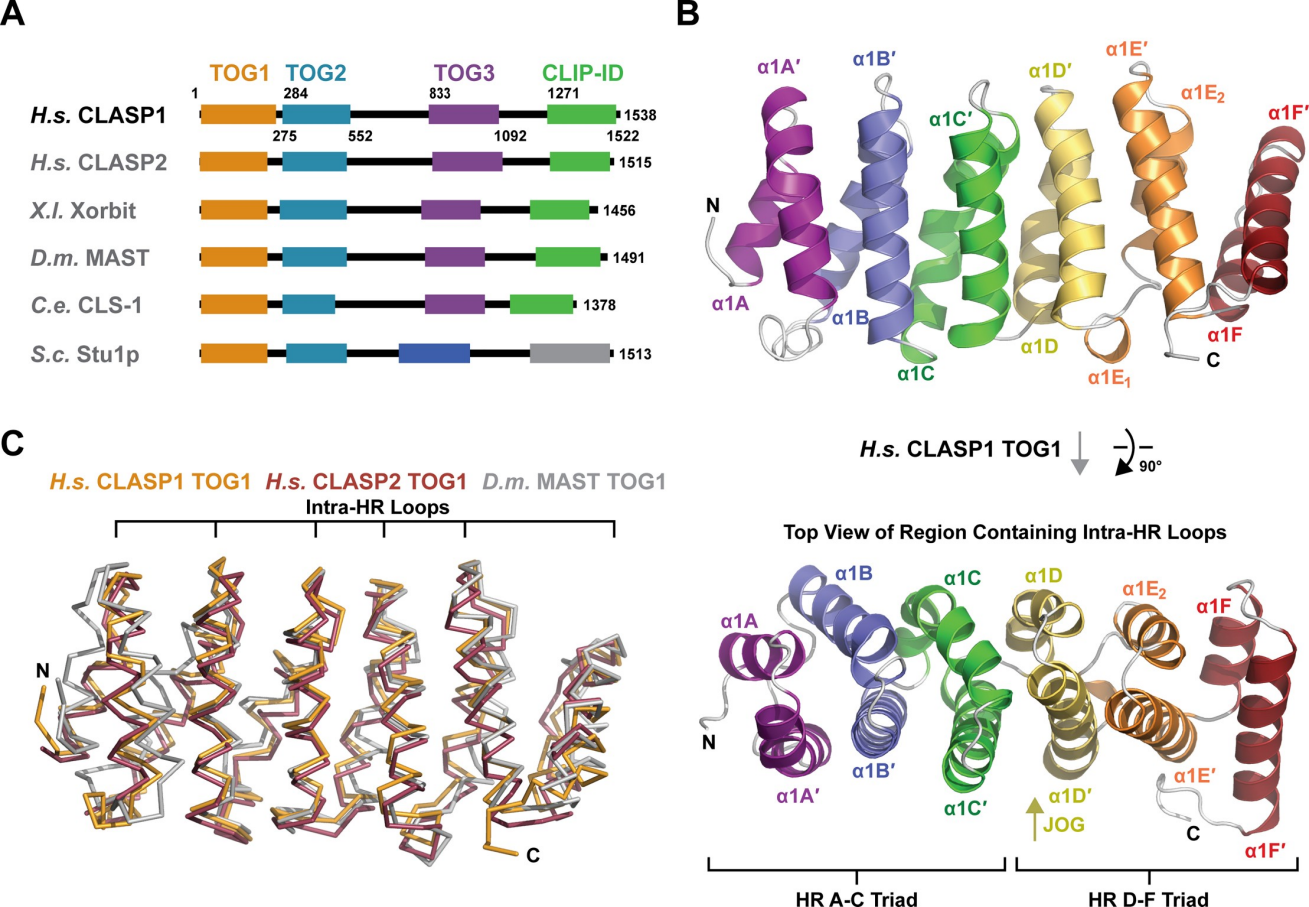
*H*. *s*. CLASP1 TOG1 forms a structurally conserved α-solenoid composed of six HRs. (A) Domain architecture of CLASP family members. *H*. *sapiens* (*H*.*s*.) CLASP1 and CLASP2, *X*. *laevis* (*X*.*l*.) Xorbit, *D*. *melanogaster* (*D*.*m*.) MAST, *C*. *elegans* (*C*.*e*.) CLS-1, and *S*. *cerevisiae* (*S*.*c*.) Stu1p. *S*.*c*. Stu1p’s two C-terminal domains are uniquely colored based on lack of homology to TOG3 and the CLIP-170 interaction domain (CLIP-ID) of animal CLASP family members. (B) Architecture of *H*.*s*. CLASP1 TOG1 shown in cartoon format. The helices of each of the six HRs (A-F) are colored across the spectrum. The image at top is rotated 90° about the x-axis to present the view shown at bottom which focuses on the intra-HR loops. N- to C-terminal directionality is shown from deep purple (N-terminus) to deep red (C-terminus). (C) Structural alignment of *H*.*s*. CLASP1 TOG1 with *H*.*s*. CLASP2 TOG1 and *D*.*m*. MAST TOG1 (PDB accession codes 5NR4 and 4G3A respectively, [25,41]).

While the CLASP and XMAP215 families differentially affect microtubule dynamics, they both employ an array of tubulin-binding TOG domains to regulate the microtubule polymer [16,22,33,34]. TOG domain structures were first determined from XMAP215 family members, revealing a 220–250 residue α-solenoid comprising six HEAT repeats (HRs) (A through F) that form a paddle-like structure [33,35]. The intra-HEAT loops that line one face of these XMAP215 family TOG domains are highly conserved and are used to engage the tubulin heterodimer [33,35]. Structural work involving TOG1 and TOG2 from the *Saccharomyces cerevisiae* (*S*.*c*.) XMAP215 family member Stu2 demonstrated that TOG domain HRs A-D and HRs E-F engage regions of β- and α-tubulin respectively that are exposed on the cytosolic surface of the microtubule [36,37]. Elucidating the structural determinants that underlie XMAP215 family TOG architecture led to the prediction and subsequent confirmation that CLASP also contains an array of cryptic TOG domains that underlies its regulatory action on microtubule dynamics [33,34]. While mammalian XMAP215 family members contain an N-terminal pentameric TOG domain array, mammalian CLASPs contains three TOG domains (TOG1-3) followed by a C-terminal CLIP-170 interaction domain (CLIP-ID) (Fig 1A) [16,22,38]. CLASP family domain organization is similar across human, *Xenopus laevis* (*X*.*l*.), *Drosophila melanogaster* (*D*.*m*.), and *Caenorhabditis elegans* (*C*.*e*.) species, but it is of note that the domain organization diverges in *S*.*c*. Stu1p, which has a TOG1 and a TOG2 domain, but its two predicted C-terminal domains lack sequence similarity to mammalian CLASP TOG3 or CLIP-ID domains. In addition, *Arabidopsis thaliana* (*A*.*t*.) CLASP lacks an apparent EB1-binding motif, though it does show enhanced microtubule plus end localization [39,40]. Furthermore, *Arabidopsis* lacks a CLIP-170 homolog, indicating that the *A*.*t*. CLASP C-terminal domain plays a role independent of CLIP-170 binding. Thus, structural and functional variation in the C-terminal domains of CLASP family members across species is predicted. For this reason, the analyses we present here focus on the TOG domains of animal CLASP family members.

TOG structures determined to date from XMAP215 and CLASP family members show dramatically different curvatures along the TOG domain’s α-solenoid axis that predict distinct tubulin-binding modes [25,26,33–38,41–44]. Of note, the structure of *H*.*s*. CLASP1 TOG2 revealed a unique bent architecture that would require a significant conformational change in either the TOG domain and/or tubulin to enable each component to fully engage [34]. While TOG domains have diverse architectures, structural data collected to date indicate that the order of these structurally distinct TOG architectures in each array is generally conserved across paralogs and species. Similarly, functional assays demonstrate that rearranging the order of these structurally distinct TOG domains within the array can compromise the collective action of the TOG array to properly regulate microtubule dynamics [38]. This has led to the hypothesis that XMAP215 and CLASP families use a common TOG array-based paradigm to regulate microtubule dynamics, but utilize distinct TOG architectures along their respective arrays to differentially regulate the polymer’s dynamics.

Structural studies of CLASP family members to date have presented the structures of *Drosophila melanogaster* (*D*.*m*.) MAST TOG1, *H*.*s*. CLASP2 TOG1, *H*.*s*. CLASP1 TOG2, *H*.*s*. CLASP2 TOG2, *S*.*c*. Stu1 TOG2, and *M*.*m*. CLASP2 TOG3 [25,26,34,41,44]. *D*.*m*. MAST TOG1 has a flat TOG architecture similar to *S*.*c*. Stu2 TOG1 [41]. Structures of *H*.*s*. CLASP1 TOG2 and *H*.*s*. CLASP2 TOG2 are similar to one another and exhibit the bent architecture described above [34,44]. The structure of *M*.*m*. CLASP2 TOG3 reveals a bent architecture, both in the plane observed in *H*.*s*. CLASP1 TOG2 and *H*.*s*. CLASP2 TOG2, as well as perpendicular to this, such that the *M*.*m*. CLASP2 TOG3 HR D-F triad likely engages unique determinants on α-tubulin [44]. A similar, yet distinct, orthogonally bent architecture was observed in structures of TOG4 from XMAP215 family members [42]. These structural findings indicate that TOG domains in the CLASP family array each have distinct architectures which may suggest distinct roles in tubulin-binding, microtubule affinity, and effects on microtubule dynamic instability. In support, recent studies of CLASP family members *H*.*s*. CLASP2 and *S*.*c*. Stu1 have assigned microtubule dynamics regulatory functions to individual CLASP family TOG domains [25,26]. Specifically for *H*.*s*. CLASP2, TOG2 and TOG3 were each found to have rescue activity and TOG2 was found to be necessary and sufficient to limit microtubule catastrophe. While significant gains have been made in elucidating CLASP family TOG structures, additional TOG structures from diverse family members are required to determine if these distinct TOG domain architectures appear in a specific, conserved order along the TOG array.

Here we structurally characterize the first two TOG domains of a mammalian CLASP, *H*.*s*. CLASP1, comparing and contrasting these structures with previously determined structures of animal CLASP family TOG domains, as well as TOG domains from the XMAP215 family of microtubule polymerases. We primarily focus our analyses on animal CLASP family members due to variation in homology, binding partners, and function noted in non-animal CLASP family members (*e*.*g*. plants and yeast). We present the X-ray crystal structure of *H*.*s*. CLASP1 TOG1 as well as a high-resolution structure of *H*.*s*. CLASP1 TOG2 (relative to our previously reported *H*.*s*. CLASP1 TOG2 structure [34]). These structures demonstrate that TOG architectures are arrayed in a specific, conserved order across the CLASP family TOG array, with each TOG domain in the array having a distinct architecture. While tubulin-binding activity has not been detected for animal CLASP family TOG1 domains [25,41,44], *H*.*s*. CLASP1 TOG1 does conform to a general tubulin-binding TOG architecture as observed in the structures of the yeast XMAP215 family member *S*.*c*. Stu2 TOG1 and TOG2 bound to tubulin [36,37,45]. However, CLASP family TOG1 domains have distinct conserved determinants that differ from the tubulin binding determinants found in XMAP215 family TOG domains that likely underlie their lack of apparent tubulin binding activity. In contrast, *H*.*s*. CLASP1 TOG2, while containing XMAP215 family TOG-like tubulin binding determinants, adheres to a convex architecture across its tubulin-binding surface that predicts a unique tubulin-binding mode. The structures of *H*.*s*. CLASP1 TOG1 and *H*.*s*. CLASP1 TOG2 are architecturally distinct from one another as well as from the previously reported structure of *M*.*m*. CLASP2 TOG3 [44]. Modeling analyses suggest that *H*.*s*. CLASP1 TOG2 and *M*.*m*. CLASP2 TOG3 each engages tubulin in the microtubule lattice in a distinct fashion. This work highlights the emerging paradigm of a structurally diverse TOG domain array in which architecturally distinct domains each play unique roles in regulating microtubule dynamics.

## Materials and methods

### Protein expression and purification

*H*.*s*. CLASP1 TOG1 (residues 1–257) and *H*.*s*. CLASP1 TOG2 (residues 284–552) bacterial expression constructs were generated using the polymerase chain reaction method and individually sub-cloned into pET28 (Millipore Sigma, Burlington, MA). *H*.*s*. CLASP1 TOG1 and TOG2 construct expression and purification protocols were identical except as noted for the growth of *H*.*s*. CLASP1 TOG1 in minimal media containing selenomethionine (to produce selenomethionine-substituted protein), and ion exchange chromatography and final exchange buffer which were optimized based on the isoelectric point of *H*.*s*. CLASP1 TOG1 (pI = 6.1), which contrasts with the isoelectric point of *H*.*s*. CLASP1 TOG2 (pI = 7.0). Constructs were transfected into *Escherichia coli* (*H*.*s*. CLASP1 TOG1: B834 methionine auxotrophic cells; *H*.*s*. CLASP1 TOG2: BL21 DE3 pLysS cells), grown to an optical density at 600 nm of 1.0 in media (*H*.*s*. CLASP1 TOG1: minimal media supplemented with seleno-L-methionine as described [46]; *H*.*s*. CLASP1 TOG2: Luria Broth) containing 50 μg/l kanamycin, the temperature lowered to 18°C, and protein expression induced with 100 μM Isopropyl β-D-1-thiogalactopyranoside for 16 hours. Cells were harvested by centrifugation, resuspended in buffer A (25 mM Tris pH 8.0, 200 mM NaCl, 10 mM imidazole, 0.1% (v/v) β-ME) at 4°C, and lysed by sonication. Phenylmethylsulfonyl fluoride was added to 1 mM final concentration. Cells debris was pelleted by centrifugation at 23,000 x g for 45 minutes and the supernatant loaded onto a 5 ml Ni^2+^-NTA column (Qiagen, Hilden, Germany). The column was washed with 500 ml buffer A and protein eluted over a 250 ml linear gradient from 100% buffer A to 100% buffer B (buffer B = buffer A supplemented with 290 mM imidazole). Peak fractions were pooled, CaCl_2_ added to 1 mM final concentration, and 0.1 mg bovine α-thrombin added to proteolytically cleave off the N-terminal His_6_ tag on each construct. After a 24 hour incubation period at 4°C, protein was filtered over 0.5 ml of benzamadine sepharose (GE Healthcare Bio-Sciences, Pittsburgh, PA) and concentrated to 1 ml in a Millipore 10k MWCO centrifugal concentrator (Millipore Sigma, Burlington, MA). *H*.*s*. CLASP1 TOG1 was diluted into 100 ml buffer C (25 mM Tris pH 8.0, 0.1% (v/v) β-ME), and loaded onto a 10 ml Q-sepharose Fast Flow column (GE Healthcare Bio-Sciences, Pittsburgh, PA). Protein was washed with 200 ml buffer C and eluted using a 250 ml linear gradient between 100% buffer C and 100% buffer D (buffer D = buffer C + 1 M NaCl). Peak fractions were pooled and protein was concentrated and exchanged into *H*.*s*. CLASP1 TOG1 storage buffer (10 mM Tris pH 8.0, 150 mM NaCl, 0.1% (v/v) β-ME) by dialyzing against 2L of storage buffer overnight at 4°C using 3.5 kDa MWCO SnakeSkin dialysis tubing (Thermo Fisher Scientific, Waltham, MA). *H*.*s*. CLASP1 TOG2 was diluted into 100 ml buffer E (25 mM Hepes pH 7.0, 0.1% (v/v) β-ME), and loaded onto a 10 ml SP-sepharose Fast Flow column (GE Healthcare Bio-Sciences, Pittsburgh, PA). Protein was washed with 200 ml buffer E and eluted using a 250 ml linear gradient between 100% buffer E and 100% buffer F (buffer F = buffer E + 1 M NaCl). Peak fractions were pooled and protein was concentrated and exchanged into *H*.*s*. CLASP1 TOG2 storage buffer (25 mM Hepes pH 7.0, 200 mM NaCl, 0.1% (v/v) β-ME).

### Crystallization, data collection, and structure determination

Selenomethionine-substituted *H*.*s*. CLASP1 TOG1 was crystallized via hanging drop: 2 μl of 10 mg/ml protein plus 2 μl of a 1 ml well solution containing 0.1 M 2-(*N*-morpholino)ethanesulfonic acid (MES) pH 6.5, 30% (v/v) PEG 600, 10% (v/v) glycerol, 18°C. Crystals were grown from microseeds that originally crystallized in 1.5 M sodium malonate, pH 6.25, 18°C. A microseed stock solution was prepared by adding 50 μL of mother liquor to the crystal drop before crushing macrocrystals using a rounded glass tip. Approximately 0.2 μl of the microseed stock solution was added to a nascent hanging drop. *H*.*s*. CLASP1 TOG2 was crystallized via hanging drop: 2 μl of 10 mg/ml protein plus 2 μl of a 1 ml well solution containing 22% (w/v) PEG 3350 and 200 mM sodium citrate (pH 8.25), 18°C. *H*.*s*. CLASP1 TOG1 and *H*.*s*. CLASP1 TOG2 crystals were transferred into the cryoprotectant oil paratone-N (Hampton Research, Aliso Viejo, CA), flash-cooled in liquid nitrogen, and diffraction data sets collected on single crystals at the Advanced Photon Source 22-ID beamline at 100 K. Data were processed using HKL2000 [47] (Table 1). Attempts to determine phases for the *H*.*s*. CLASP1 TOG1 structure using single wavelength anomalous diffraction (SAD) phasing methods failed from crystals grown in 1.5 M sodium malonate, pH 6.25. Attempts to obtain phasing via molecular replacement (TOG1 search model: *D*.*m*. MAST TOG1, PDB accession code 4G3A, chain A [41]) also failed. Thus, crystals grown in 0.1 M MES pH 6.5, 30% (v/v) PEG 600, 10% (v/v) glycerol, seeded from the original crystals grown in sodium malonate, were used for anomalous diffraction experiments and provided the SAD phasing for structure determination. SAD phasing was performed using phenix.autosol (PHENIX) [48], which identified 14 Se atoms at a figure of merit of 0.440. Density modification was carried out using RESOLVE [49], and identified 2 copies for NCS averaging (7 Se atoms per molecule) to improve electron density maps. The *H*.*s*. CLASP1 TOG2 structure was determined via molecular replacement using a search model: *H*.*s*. CLASP1 TOG2, PDB accession code 4K92, chain A [34].

**Table 1:** Data processing and refinement statistics.

Crystal	*H*.*s*. CLASP1 TOG1	*H*.*s*. CLASP1 TOG2
**Data Collection**		
Wavelength (Å)	0.97964	1.0000
Space group	P2_1_	P2_1_
Cell dimensions: a,b,c (Å); β (°)	41.0, 114.0, 99.9; 99.8	51.6, 67.4, 81.0; 98.3
Resolution (Å)	50.0–2.15 (2.23–2.15)	50.0–1.78 (1.84–1.78)
# Reflections: Measured / Unique	148,462 (11,001) / 41,883 (3,667)	319,655 (19,398) / 51,928 (4,492)
Completeness (%)	92.3 (81.2)	98.1 (85.4)
Mean redundancy	3.5 (3.0)	6.8 (4.3)
<I/σI>	9.6 (1.96)	15.1 (2.8)
R_sym_	0.155 (0.646)	0.095 (0.278)
**Refinement**		
Resolution (Å)	32.95–2.15 (2.20–2.15)	38.1–1.78 (1.83–1.78)
R/ R_free_ (%)	19.0 (24.4) / 24.4 (33.3)	17.7 (29.5) / 22.1 (38.0)
# Reflections, R/R_free_	21,034 (1768) / 1930 (159)	48317 (3683) / 1874 (153)
Total atoms: Protein / Water	3,685 / 183	3,920 / 659
Stereochemical ideality (rmsd): Bonds / Angles (Å/°)	0.011 / 1.38	0.008 / 1.05
Mean B-factors (Å^2^): Overall / Protein / Water	28.8 / 28.6 / 31.3	29.0 / 27.1 / 39.9
Ramachandran Analysis: Favored / Allowed (%)	98.1 / 1.7	99.2 / 0.8
**PDB accession code**	6MQ5	6MQ7

Values in parentheses indicate statistics for the highest-resolution shell.

Initial models were built using AutoBuild (PHENIX) followed by reiterative buildings in Coot [50] and subsequent refinement runs using phenix.refine (PHENIX) [48]. Refinement runs used real space, simulated annealing refinement protocols (temperatures: 5,000 K start, 300 K final, 50 steps), and individual B-factor refinement, using a maximum-likelihood target. TLS groups were assigned using PHENIX and TLS and individual B-factor refinement was used in later stages of refinement. The final refinement runs produced an R_free_ value of 24.4% for the *H*.*s*. CLASP1 TOG1 structure and an R_free_ value of 22.1% for the *H*.*s*. CLASP1 TOG2 structure. The final *H*.*s*. CLASP1 TOG1 model includes residues 1–234 for chain A, residues 1–236 for chain B, and 183 water molecules. The final *H*.*s*. CLASP1 TOG2 model includes residues 295–538 for chains A and B and 659 water molecules. Water molecules were validated through inspection of electron density and hydrogen bonding distances. Data collection and refinement statistics are summarized in Table 1.

### Structure analyses

Structure images were generated using the PyMOL Molecular Graphics System, version 1.5.0.5 (Schrödinger, LLC, New York, NY). Electrostatic calculations used the PyMOL plugin APBS [51]. Pairwise structure comparisons and root mean square displacement (rmsd) values were calculated using the Dali server [52]. CLASP family TOG domains were modeled on the structure of tubulin using the following protocol: the first HR triad (HRs A-C) of *H*.*s*. CLASP1 TOG1, *H*.*s*. CLASP1 TOG2, and *M*.*m*. CLASP2 TOG3 were structurally aligned to the first HR triad of *S*.*c*. Stu2 TOG2 from the *S*.*c*. Stu2 TOG2-tubulin complex structure (PDB accession code 4U3J, [37]) using the Dali server [52] and the resulting output used as a reference point for superpositioning the full, respective CLASP TOG domain onto the tubulin coordinates from the 4U3J structure. To model *M*.*m*. CLASP2 TOG3 on the microtubule lattice, the model of *M*.*m*. CLASP2 TOG3 bound to a tubulin heterodimer (described above) was superimposed onto the microtubule lattice coordinates of GMPCPP-bound tubulin (PDB accession code 3JAT [53]) using tubulin heterodimer coordinates from each model as input to the Dali server. The positioning of the *M*.*m*. CLASP2 TOG3 domain relative to the tubulin heterodimers from the 3JAT structure was then analyzed. Similarity values for the sequence alignments presented were calculated using the following amino acid similarity criteria (amino acids in parentheses were classified as similar): (QN), (RK), (ILV), (DE), (TSC), (GA), (FYW).

## Results and discussion

### The α-solenoid HEAT repeat structure of *H*.*s*. CLASP1 TOG1

To determine the structure of *H*.*s*. CLASP1 TOG1 we crystallized a selenomethionine-substituted construct embodying residues 1 to 257 and collected a 2.15 Å resolution single wavelength anomalous diffraction dataset. Crystals belong to the space group P2_1_ and contain two protomers in the asymmetric unit. The data was 92.3% complete overall and 81.2% complete in the high-resolution shell (2.23–2.15 Å) with an average I/σI of 1.96 in the high-resolution shell. The structure was refined to R and R_free_ values of 19.0% and 24.4% respectively. The difference between the R and R_free_ values likely reflects mild structural variability in the domain’s terminal regions (as indicated by higher B-factors in these regions). At the C-terminal region this leads to the lack of apparent electron density for the C-terminal 21 residues in the *H*.*s*. CLASP1 TOG1 construct which were not modeled. However the difference between the R and R_free_ values is similar to those reported for other CLASP family TOG domain structures [26,44]. Data collection and refinement statistics are presented in Table 1.

*H*.*s*. CLASP TOG1 is an α-solenoid structure approximately 65 Å in length, consisting of six HRs designated HR A through F (Fig 1B). We delineate the helices of each HR α and α’, followed by the number of the TOG domain in the array and the letter of the HR to which the helix belongs. The HRs conform to a general TOG-domain architecture. The first HR triad (HRs A-C) has a right-handed twist (if viewed along the axis of the α-solenoid from HR A to HR C, each subsequent HR is rotated clockwise relative to the HR that preceded it). The second HR triad (HRs D-F) is translated relative to the axis of the first HR triad, introducing a jog in the α-solenoid that gives the domain a flat, paddle-like architecture, rather than an elongated spiral common to other α-solenoid structures. HRs D and E are oriented with a right-handed twist relative to one another, while HRs E and F are oriented with a left-handed twist (Fig 1B, lower panel). The architecture of *H*.*s*. CLASP1 TOG1 is similar to that of *H*.*s*. CLASP2 TOG1 and *D*.*m*. MAST TOG1 [25,41], with overall pairwise Cα rmsd values of 1.2 and 2.4 Å for the *H*.*s*. CLASP1-*H*.*s*. CLASP2 and *H*.*s*. CLASP1-*D*.*m*. MAST comparisons respectively (Fig 1C, Table 2, calculated using the Dali server [52]). *H*.*s*. CLASP1 TOG1 structurally aligns to *D*.*m*. MAST TOG1 (the more divergent comparison) best across the TOG face composed of intra-HR loops.

**Table 2:** CLASP family TOG domain pairwise structural comparison.

Protein Aligned #1 (PDB code/chain)	Protein Aligned #2 (PDB code/chain)	Region Aligned	#Cα Atoms Aligned	rmsd (Å)	% Identity	Z-Score
*H*.*s*. CLASP1 TOG1 (6MQ5/A)	*H*.*s*. CLASP1 TOG1 (6MQ5/B)	Full Domain	234/236	0.9	100	36.9
*H*.*s*. CLASP1 TOG1 (6MQ5/B)	*H*.*s*. CLASP2 TOG1 (5NR4/A)	Full Domain	220/220	1.2	61	31.2
*H*.*s*. CLASP1 TOG1 (6MQ5/B)	*D*.*m*. MAST TOG1 (4G3A/A)	Full Domain	224/227	2.4	37	27.3
*H*.*s*. CLASP1 TOG2 (6MQ7/A)	*H*.*s*. CLASP1 TOG2 (6MQ7/B)	Full Domain	224/224	1.4	100	38.7
*H*.*s*. CLASP1 TOG2 (6MQ7/A)	*H*.*s*. CLASP1 TOG2 (4K92/B)	Full Domain	243/243	0.3	100	40.7
*H*.*s*. CLASP1 TOG2 (6MQ7/B)	*H*.*s*. CLASP1 TOG2 (4K92/B)	Full Domain	243/243	1.3	100	38.5
*H*.*s*. CLASP1 TOG2 (6MQ7/A)	*H*.*s*. CLASP2 TOG2 (3WOY/A)	Full Domain	244/246	1.3	81	37.9
*H*.*s*. CLASP1 TOG2 (6MQ7/B)	*H*.*s*. CLASP2 TOG2 (3WOY/A)	Full Domain	244/246	1.0	81	37.9
*H*.*s*. CLASP1 TOG1 (6MQ5/B)	*H*.*s*. CLASP1 TOG2 (6MQ7/A)	Full Domain	212/222	3.3	12	19.9
*H*.*s*. CLASP1 TOG1 (6MQ5/B)	*H*.*s*. CLASP1 TOG2 (6MQ7/A)	HR ABC	114/123	2.2	12	14.4
*H*.*s*. CLASP1 TOG1 (6MQ5/B)	*H*.*s*. CLASP1 TOG2 (6MQ7/A)	HR DEF	97/99	2.3	14	12.4
*H*.*s*. CLASP1 TOG1 (6MQ5/B)	*M*.*m*. CLASP2 TOG3 (3WOZ/A)	Full Domain	213/225	2.9	13	18.8
*H*.*s*. CLASP1 TOG1 (6MQ5/B)	*M*.*m*. CLASP2 TOG3 (3WOZ/A)	HR ABC	112/114	1.9	15	14.9
*H*.*s*. CLASP1 TOG1 (6MQ5/B)	*M*.*m*. CLASP2 TOG3 (3WOZ/A)	HR DEF	97/109	2.6	11	10.0
*H*.*s*. CLASP1 TOG2 (6MQ7/A)	*M*.*m*. CLASP2 TOG3 (3WOZ/A)	Full Domain	206/225	3.4	12	17.9
*H*.*s*. CLASP1 TOG2 (6MQ7/A)	*M*.*m*. CLASP2 TOG3 (3WOZ/A)	HR ABC	111/114	2.2	13	13.9
*H*.*s*. CLASP1 TOG2 (6MQ7/A)	*M*.*m*. CLASP2 TOG3 (3WOZ/A)	HR DEF	94/109	2.9	13	9.9

### *H*.*s*. CLASP1 TOG1 is highly conserved across the surface defined by intra-HR loops

**Fig 2:**
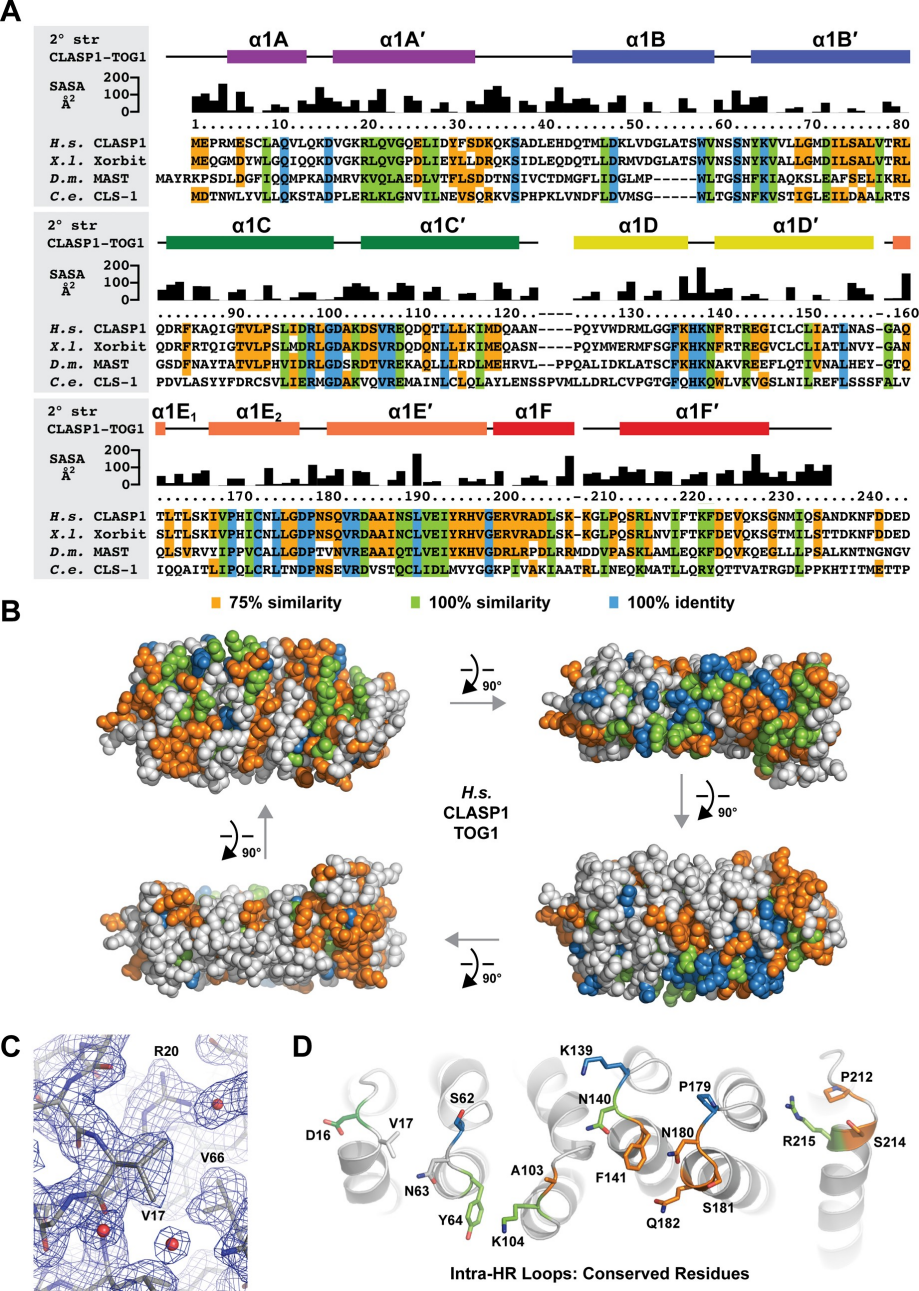
*H*.*s*. CLASP1 TOG1 has a conserved face delineated by intra-HR loops. (A) Sequence alignment of CLASP family TOG1 domains from *H*. *sapiens (H*.*s*.*)* CLASP1, *X*. *laevis* (*X*.*l*.*)* Xorbit, *D*. *melanogaster (D*.*m*.*)*. MAST, and *C*. *elegans (C*.*e*.*)* CLS-1. *H*.*s*. CLASP1 TOG1 2° structure and solvent accessible surface area (SASA) are shown above the alignment. Rectangles represent α-helices. Residues are highlighted based on 100% identity (blue), 100% similarity (light green), and 75% similarity (orange)(see Materials and methods for amino acid similarity criteria). (B) Cross-species conservation delineated in A, mapped on the *H*.*s*. CLASP1 TOG1 structure, and rotated in 90° steps about the x-axis. The orientation at upper left corresponds to the upper orientation in Fig 1B. (C) View of the HR A loop residue V17 shown in stick format with 2mF_o_-DF_c_ electron density shown in blue, contoured at 1.0 σ. (D) Intra-HR residues shown in stick format with conservation color-coded as in A and B.

To examine *H*.*s*. CLASP1 TOG1 surface residue conservation, we generated a sequence alignment involving CLASP family members *H*.*s*. CLASP1, *X*.*l*. Xorbit, *D*.*m*. MAST, and *C*.*e*. CLS-1. We contoured conservation at 100% identity (blue), 100% similarity (light green), and 75% similarity (orange)(see Materials and methods for amino acid similarity criteria) and mapped this scheme on the *H*.*s*. CLASP1 TOG1 structure (Fig 2A and 2B). The domain face formed by intra-HR loops displayed the highest degree of conservation (Fig 2B, upper right), with additional conservation mapping to the face formed by the α’ helices of each HR (Fig 2B, upper left). TOG domains of the XMAP215 family are known to engage tubulin using the intra-HR loop surface. While *H*.*s*. CLASP1 TOG1 has a subset of intra-HR residues that are positionally similar to those found in XMAP215 family TOG tubulin binding determinants, there are also significant differences. Specifically, XMAP215 family TOG domains primarily contain a tryptophan in the HR A loop. The homologous position in *H*.*s*. CLASP1 TOG1 is a valine (V17) that is not conserved (Fig 2C and 2D); *D*.*m*. MAST TOG1 and *C*.*e*. CLS-1 TOG1 have a methionine and proline in the equivalent position respectively (Fig 2A). Interestingly, while XMAP215 family TOG domains are primarily conserved across the HR A-C intra-HEAT loops, animal CLASP family TOG1 conservation is primarily focused along the surface formed by the HR C-E intra-HR loops. Collectively, *H*.*s*. CLASP1 TOG1 has a canonical TOG domain architecture, but its unique surface residue conservation pattern is distinct and may align with a specific non-tubulin binding role for a TOG domain such as autoregulation, as has been ascribed for *H*.*s*. CLASP2 TOG1 [25].

### Mammalian *H*.*s*. CLASP1 TOG2 forms a conserved convex architecture

Previous structural work analyzing *H*.*s*. CLASP1 TOG2 and *H*.*s*. CLASP2 TOG2 revealed a highly bent, convex TOG architecture [34,44]. To determine if this architecture was due to crystal packing, we determined the structure of *H*.*s*. CLASP1 TOG2 in a different space group, P2_1_, as compared to the initial structure that was determined in the space group P2_1_2_1_2_1_ [34]. Native diffraction data was collected to a resolution of 1.78 Å, was 98.1% complete overall, and was 85.4% complete in the 1.84–1.78 Å high-resolution shell with an average I/σI of 2.8 in the high-resolution shell. The crystal contains two protomers in the asymmetric unit. The structure was solved by molecular replacement using the structure of *H*.*s*. CLASP1 TOG2 as a search model (PDB accession code 4K92 [34]). The structure was refined to R and R_free_ values of 17.7% and 22.1% respectively. The difference between the R and R_free_ value likely reflects mild structural variability in the domain’s terminal regions (as indicated by higher B-factors in these regions). This led to the lack of apparent electron density for the N-terminal 11 residues and the C-terminal 14 residues in the *H*.*s*. CLASP1 TOG2 construct which were not modeled in the structure. However the difference between the R and R_free_ values is similar to those reported for other CLASP family TOG domain structures [26,34,44]. Data collection and refinement statistics are presented in Table 1. In comparison, the previously determined *H*.*s*. CLASP1 TOG2 structure (space group P2_1_2_1_2_1_, PDB accession code 4K92) was determined to a resolution of 2.00 Å, the data was 92.3% complete overall, was 64.9% complete in the 2.07–2.00 high-resolution shell, and had an average I/σI of 1.96 in the high-resolution shell [34].

**Fig 3:**
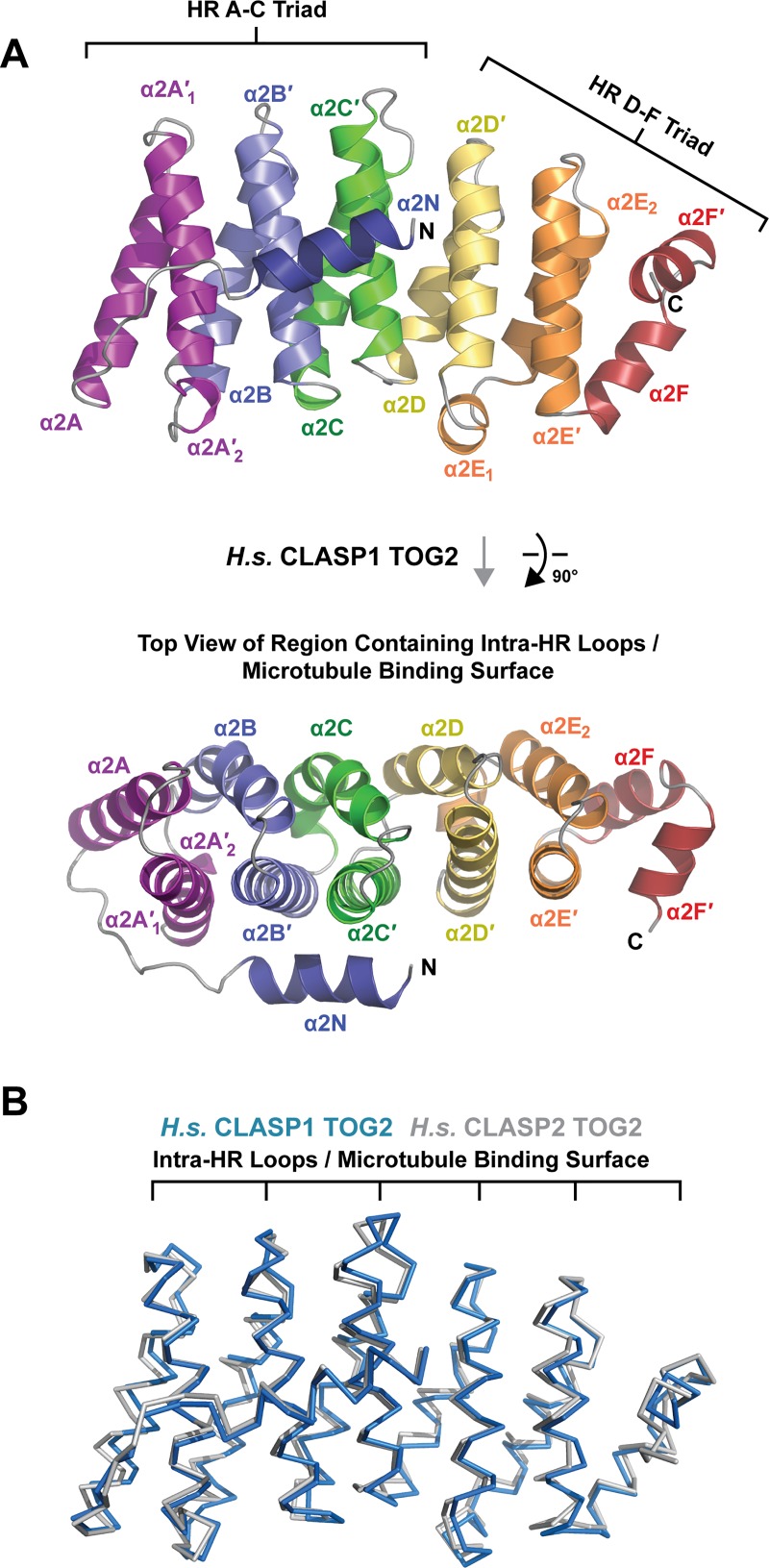
*H*.*s*. CLASP1 TOG2 forms a convex α-solenoid, structurally homologous to *H*.*s*. CLASP2 TOG2. (A) Architecture of *H*.*s*. CLASP1 TOG2 shown in cartoon format. The helices of each of the six HRs (A-F) are colored across the spectrum. The image at top is rotated 90° about the x-axis to present the view shown at bottom which focuses on the intra-HR loops. N- to C-terminal directionality across the HR α-solenoid is shown from deep purple (N-terminus) to deep red (C-terminus), with the N-terminal α2N helix colored blue. (B) Structural alignment of *H*.*s*. CLASP1 TOG2 and *H*.*s*. CLASP2 TOG2 (PDB accession code 3WOY, [44]) using the Dali server [52].

The *H*.*s*. CLASP1 TOG2 structure determined in space group P2_1_ conforms to the bent, α-solenoid TOG architecture observed in space group P2_1_2_1_2_1_ (Fig 3A). As previously observed, *H*.*s*. CLASP1 TOG2 is 65 Å in length with a bend between the HR A-C and HR D-F triads that orients the HR D-F intra-HR loop surface at ~30° relative to the plane established by the HR A-C intra-HR loops. This deviates significantly from the flat surface observed across XMAP215 family TOG intra-HR loops used to engage tubulin (discussed further below). In addition to its bent architecture, *H*.*s*. CLASP1 TOG2 has a conserved N-terminal helix, α2N, which is positioned alongside, and orthogonal to the α2B’ and α2C’ helices (Fig 3A). The two protomers in the P2_1_ asymmetric unit have an overall Cα rmsd of 1.4 Å (Table 2). Comparing protomers determined in the P2_1_ space group to those determined in the P2_1_2_1_2_1_ space group showed little structure deviation with low overall Cα pairwise rmsd values that ranged from 0.3 to 1.3 Å (Table 2). Comparison of the *H*.*s*. CLASP1 TOG2 protomers with the structure of *H*.*s*. CLASP2 TOG2, which has 81% sequence identity, yielded overall Cα rmsd values that ranged from 1.0–1.3 Å (Fig 3B, Table 2). Collectively, the bent architecture of CLASP family TOG2 domains is conserved and reflects a key structural state of the domain.

### *H*.*s*. CLASP1 TOG2 is highly conserved across the convex intra-HR loop surface

**Fig 4:**
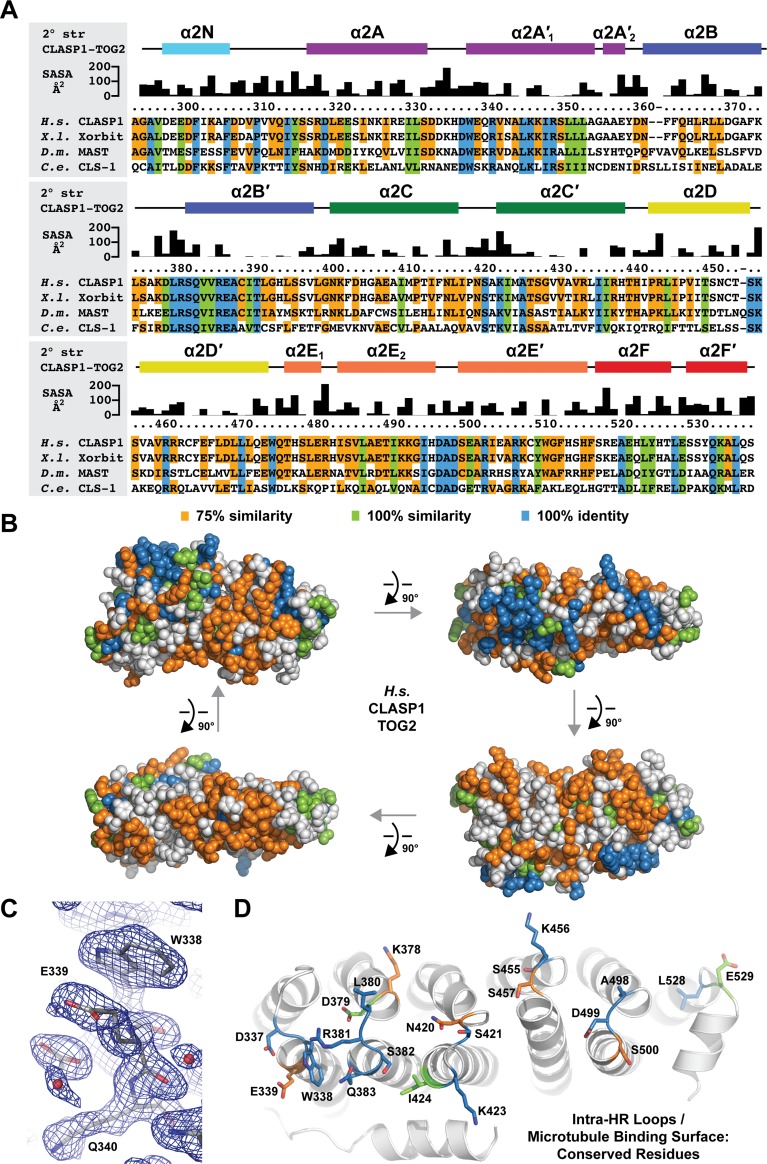
*H*.*s*. CLASP1 TOG2 has a conserved face delineated by intra-HR loops. (A) Sequence alignment of CLASP family TOG2 domains from *H*. *sapiens (H*.*s*.*)* CLASP1, *X*. *laevis (X*.*l*.*)* Xorbit, *D*. *melanogaster (D*.*m*.*)* MAST, and *C*. *elegans (C*.*e*.*)*. CLS-1. *H*.*s*. CLASP1 TOG2 2° structure and solvent accessible surface area (SASA) are shown above the alignment. Rectangles represent α-helices. Residues are highlighted based on 100% identity (blue), 100% similarity (light green), and 75% similarity (orange)(see Materials and methods for amino acid similarity criteria). (B) Cross-species conservation delineated in A, mapped on the *H*.*s*. CLASP1 TOG2 structure and rotated in 90° steps about the x-axis. The orientation at upper left corresponds to the upper orientation in Fig 3A. (C) View of the HR A loop residue W338 shown in stick format with 2mF_o_-DF_c_ electron density shown in blue, contoured at 1.0 σ. (D) Intra-HR residues shown in stick format with conservation color-coded as in A and B.

We next examined *H*.*s*. CLASP1 TOG2 surface residue conservation using the same species and conservation criteria laid forth in our *H*.*s*. CLASP1 TOG1 analysis (Fig 4A and 4B). As observed with *H*.*s*. CLASP1 TOG1, the domain face formed by intra-HEAT loops displayed the highest degree of conservation (Fig 4B, upper right), with additional conservation mapping to the orthogonally-positioned α2N helix (Fig 4B, upper left). A significant amount of surface residue conservation mapped to the remaining faces of the domain, pertaining primarily to the 75% similarity criteria. Of the highly conserved intra-HR loops, most of the conservation is located along the first triad, HR A-C. The HR A loop contains a conserved tryptophan, positioned equivalent to the conserved HR A loop tryptophan found in XMAP215 family TOG domains that engages β-tubulin (Fig 4C and 4D) [33,35–37]. A significant, yet lower degree of conservation is located along the second triad’s (HR D-F) intra-HR loops.

### The CLASP family TOG array is structurally diverse

**Fig 5:**
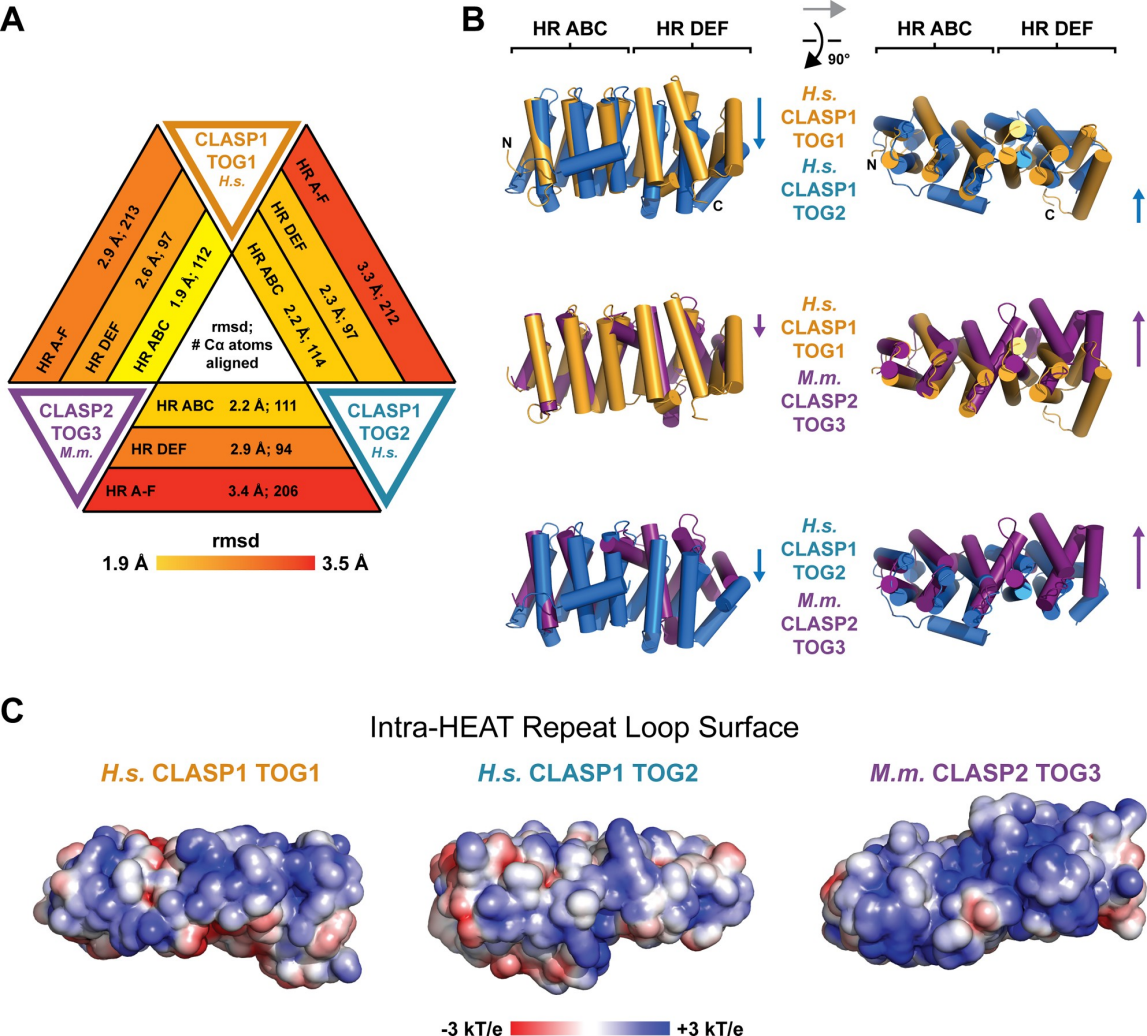
CLASP family TOG1, TOG2, and TOG3 domains each have unique architectures. (A) Comparison of *H*.*s*. CLASP1 TOG1, *H*.*s*. CLASP1 TOG2, and *M*.*m*. CLASP2 TOG3 (PDB accession code 3WOZ, [44]) structures using rmsd analysis of corresponding Cα atoms across the three domains (Table 2, Dali server [52]). Pairwise analysis was performed, comparing HRs A-C, D-F, and all six HRs: A-F. (B) Pairwise structural comparison of CLASP family TOGs 1–3. The *H*.*s*. CLASP1 TOG2 and *M*.*m*. CLASP2 TOG3 HR A-C triad was structurally aligned to the *H*.*s*. CLASP1 TOG1 HR A-C triad using the Dali server and respective pairwise comparisons generated [52]. Images at left are oriented with each domain’s intra-HR loops positioned at the top of the domain as shown. The images at right were produced after a 90° rotation about the x-axis and focus on the respective surfaces composed of intra-HR loops. (C) Electrostatic surface potential mapped on the structures of *H*.*s*. CLASP1 TOG1, *H*.*s*. CLASP 1 TOG2, and *M*.*m*. CLASP2 TOG3. The surface of each domain presented is the surface composed of intra-HR loops, oriented as presented in the images at right in panel B.

We next analyzed structural diversity across the CLASP family TOG array, comparing our structures of *H*.*s*. CLASP1 TOG1, *H*.*s*. CLASP1 TOG2, as well as the previously reported structure of *M*.*m*. CLASP2 TOG3 (the *M*.*m*. CLASP2 TOG3 structure was used for comparative analysis because no structure of *H*.*s*. CLASP1 TOG3 has been reported yet). We used the Dali server to structurally align the domains and calculate and overall rmsd value for corresponding Cα atoms [52]. Comparative analysis yielded the following high rmsd values: *H*.*s*. CLASP1 TOG1 versus *H*.*s*. CLASP1 TOG2: 3.3 Å rmsd; *H*.*s*. CLASP1 TOG1 versus *M*.*m*. CLASP2 TOG3: 2.9 Å rmsd; and *H*.*s*. CLASP1 TOG2 versus *M*.*m*. CLASP2 TOG3: 3.4 Å rmsd (Fig 5A, Table 2). To determine whether specific subdomains contributed to this structural diversity, we again used the Dali server and analyzed the Cα rmsd across the TOG domains for each HR triad: HR A-C and HR D-F. The HR A-C triads aligned best, with rmsd values ranging from 1.9 Å (*H*.*s*. CLASP1 TOG1 versus *H*.*s*. CLASP2 TOG3) to 2.2 Å (*H*.*s*. CLASP1 TOG1 and *M*.*m*. CLASP2 TOG3 versus *H*.*s*. CLASP1 TOG2)(Fig 5A, Table 2). In contrast, the HR D-F triads had a higher degree of structural variance, with rmsd values ranging from 2.3 Å (*H*.*s*. CLASP1 TOG1 versus *H*.*s*. CLASP1 TOG2) to 2.9 Å (*H*.*s*. CLASP1 TOG2 versus *M*.*m*. CLASP2 TOG3)(Fig 5A, Table 2). Thus, while the first triad is structurally conserved across the CLASP family TOG array, the second triad exhibits a higher degree of structural diversity. To determine if the relative positioning of the triads in each TOG domain also contributes to structural diversity across the CLASP family TOG array, we superimposed the full TOG domains, inputting the Cα coordinates from each TOG domain’s respective HR A-C triad to the Dali server as alignment determinants (Fig 5B). This set the HR A-C triad as a reference point for comparative analysis of the positioning of each TOG domain’s HR D-F triad. While *H*.*s*. CLASP1 TOG1 is relatively flat across the intra-HR loop surface, the alignment highlighted the relative bend between *H*.*s*. CLASP1 TOG2’s two triads that angles HR D-F downwards (Fig 5B, top panel). While *M*.*m*. CLASP2 TOG3 is flat across the HR A-E intra-HR loop surface, the HR F intra-HR loop region is positioned downward from the HR A-E intra-HR loop surface. When the intra-HR loop surfaces of *M*.*m*. CLASP2 TOG3 are viewed from above (Fig 5B, right panel), additional shifts (orthogonal to the relative bend observed in *H*.*s*. CLASP1 TOG2) are evident. While *H*.*s*. CLASP1 TOG1 bends to the side of the domain defined by the HR α’ helices, *H*.*s*. CLASP1 TOG2 is relatively straight. In contrast, *M*.*m*. CLASP2 TOG3 bends in the opposite direction, towards the side of the domain defined by the HR α helices. Collectively, the CLASP family TOG array is structurally diverse, driven primarily by architectural diversity in the HR D-F triads as well as the relative positioning of the triads in the respective TOG domain. While each TOG domain in the array is architecturally distinct, the intra-HR loop surface of each domain is dominated by basic electrostatics and hydrophobic content (Fig 5C).

### Varied CLASP family TOG architectures suggest distinct tubulin binding modes

**Fig 6:**
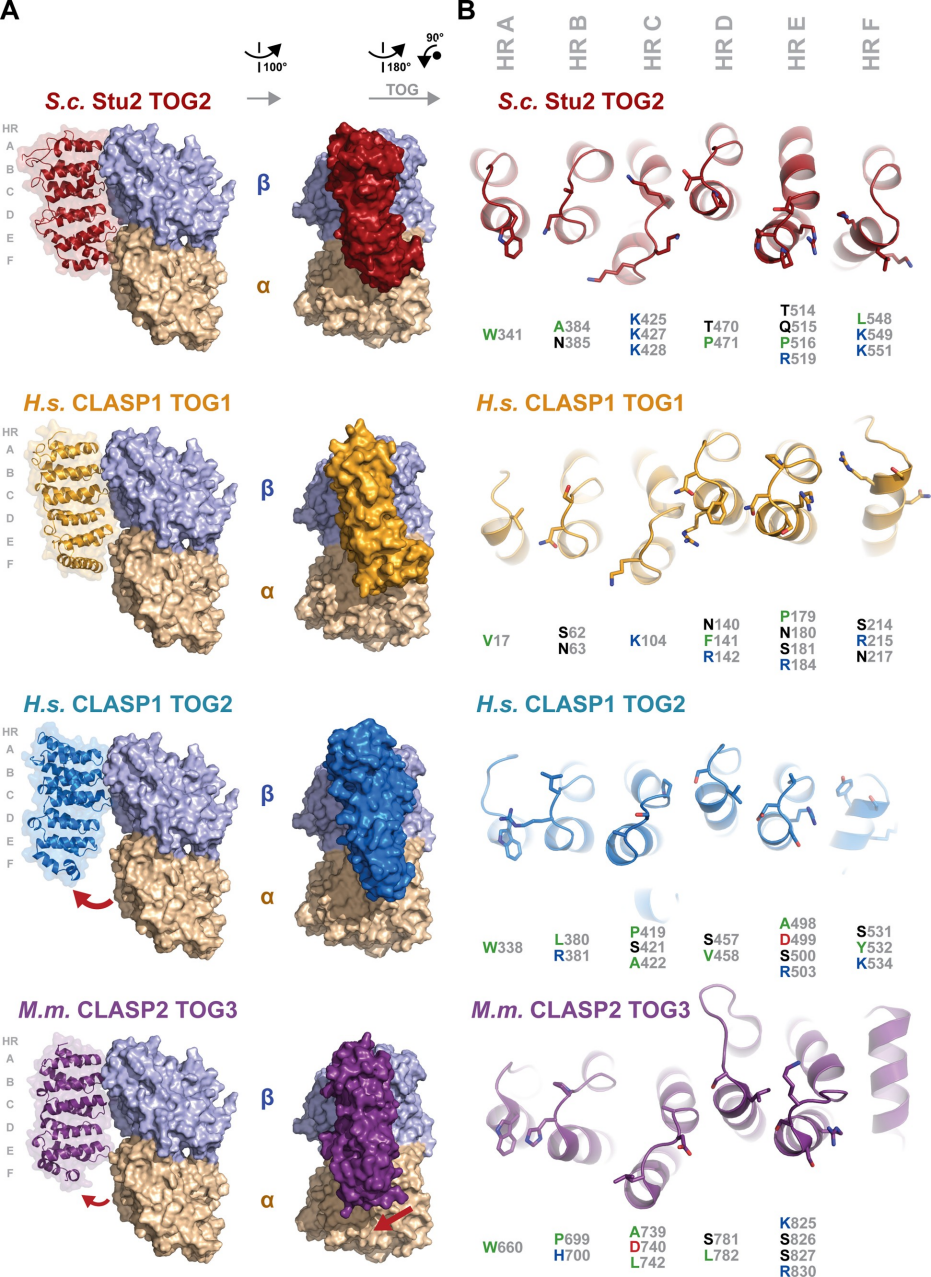
Distinct CLASP family TOG domain architectures predict distinct tubulin binding properties. (A) CLASP family TOG domains modeled on tubulin based on the structure of the XMAP215 microtubule polymerase family member *S*.*c*. Stu2 TOG2 in complex with αβ-tubulin (PDB accession code 4U3J [37], shown at top; *S*.*c*. Stu2 TOG2 in red, α- and β-tubulin shown in wheat and lavender respectively). To generate models of CLASP family TOG domains bound to tubulin in a similar mode, the first HR triad (A-C) from each of the CLASP family TOG domain structures analyzed was structurally aligned to the *S*.*c*. Stu2 TOG2 HR A-C triad using the Dali server [52]. TOG domains at left are shown in cartoon format along with a transparent molecular envelope. The images at right were generated after a 90° rotation about the y-axis and depict each TOG domain in surface representation. The *M*.*m*. CLASP2 TOG3 structure is from PDB accession code 3WOZ [44]. (B) Comparative analysis of the intra-HR loop residues of each of the CLASP family TOG domains analyzed, which in *S*.*c*. Stu2 TOG2 are used to bind tubulin. The orientation of each domain, relative to the orientation shown in A (right panel) was generated after a 180° rotation about the y-axis, followed by a 90°Counterclockwise rotation about the z-axis.

While structures of XMAP215 family TOG domains from the yeast member *S*.*c*. Stu2 bound to tubulin have informed how these TOG domains engage tubulin [36,37], how CLASP family TOG domains bind lattice-incorporated tubulin to regulate the polymer’s dynamics remains unknown. Studies to date have implicated animal CLASP family TOG2 and TOG3 domains in microtubule-binding, however, binding between animal CLASP family TOG1 domains and tubulin (free or lattice bound) has not been detected [33,34,41,44,45]. To gain insight into CLASP family TOG-tubulin binding modes and the potential basis for the lack of detectable CLASP family TOG1-tubulin binding, we superimposed CLASP family TOG domains on the *S*.*c*. Stu2 TOG2-tubulin structure and analyzed the modeled complexes. We used the *H*.*s*. CLASP1 TOG1 and *H*.*s*. CLASP1 TOG2 structures reported here as well as the previously reported *M*.*m*. CLASP2 TOG3 structure [44], the only CLASP family TOG3 structure currently available for the analysis. To generate these models, we aligned CLASP family TOG domains to *S*.*c*. Stu2 TOG2 by superpositioning the first HR triads of each domain using the Dali server [52]. The basis for this was the structural conservation noted across the first HR A-C triad, which extended to *S*.*c*. Stu2 TOG2’s first triad. *S*.*c*. Stu2 TOG2 engages the curved state of the αβ-tubulin heterodimer, which reflects tubulin’s free state found in solution and at polymerizing and depolymerizing microtubule plus ends, as compared to the straight conformation found along the length of a microtubule [36,54,55]. *S*.*c*. Stu2 TOG2 HRs A-D engage β-tubulin while HRs E-F engage α-tubulin (Fig 6A) [36]. Key *S*.*c*. Stu2 TOG2-tubulin interaction determinants are a HR A loop tryptophan, an alanine and asparagine in the HR B loop, basic residues in the HR C, E, and F loops, and a threonine and proline residue in the HR D loop (Fig 6B). While animal CLASP family TOG1 domains do not have reported tubulin-binding activity, *H*.*s*. CLASP1 TOG1 superimposes on the Stu2 TOG2-αβ-tubulin structure surprisingly well, with its intra-HEAT loops positioned to complement the surface of the tubulin heterodimer. We next compared intra-HR tubulin-binding determinants in *S*.*c*. Stu2 TOG2 to their positional counterparts in *H*.*s*. CLASP1 TOG1 to see what differences might underlie the domain’s lack of detectable tubulin and/or microtubule binding activity. Analysis shows that *H*.*s*. CLASP1 TOG1 has numerous residues that differ from the position-equivalent tubulin-binding determinants in *S*.*c*. Stu2 TOG2. *H*.*s*. CLASP1 TOG1 lacks the HR A loop tryptophan commonly found in XMAP215 family TOG domains (W341 in *S*.*c*. Stu2 TOG2). Instead, *H*.*s*. CLASP1 TOG1 has a valine (V17) at the equivalent position. The *H*.*s*. CLASP1 TOG1 HR B loop has a serine (S62) in place of the alanine (A384) found in *S*.*c*. Stu2 TOG2. The *H*.*s*. CLASP1 TOG1 HR C loop lacks many of the basic residues found in *S*.*c*. Stu2 TOG2 HR C. In the HR D loop, *H*.*s*. CLASP1 TOG1 has an asparagine (N140) and a bulky phenylalanine (F141) that contrast respectively with the threonine (T470) and proline (P471) of *S*.*c*. Stu2 TOG2. The residue composition and relative arrangement of the loops in HRs E and F also differ significantly between *H*.*s*. CLASP1 TOG1 and *S*.*c*. Stu2 TOG2. Overall, while *H*.*s*. CLASP1 TOG1 has an architecture that aligns well with the tubulin-binding TOG domains of the XMAP215 family, many of the intra-HR tubulin binding determinants found in XMAP215 family TOG domains are not present in *H*.*s*. CLASP1 TOG1. This overall difference in the composition of the intra-HR loops likely explains the lack of tubulin and microtubule binding activity reported for animal CLASP family TOG1 domains. Specifically, in vitro microtubule binding co-sedimentation assays using *D*.*m*. MAST TOG1 failed to find significant microtubule binding activity [41]. Likewise, isothermal titration calorimetry assays and in vitro microtubule co-sedimentation assays failed to detect significant *H*.*s*. CLASP2 TOG1 binding to tubulin and microtubules respectively [25]. Work analyzing yeast (*S*.*c*.) Stu1 TOG1 failed to find free tubulin binding activity using in vitro as well as extract co-purification assays [26,45]. However, while studies of the CLASP family TOG1 domain have failed to detect tubulin and microtubule binding, it is possible that TOG1 may exert weak tubulin-binding activity in the context of a larger construct. For example, a construct embodying *S*.*c*. Stu1 TOG1 and part of the linker bridging TOG1 and TOG2 (but lacking TOG2) displayed weak microtubule binding activity (40 μM) in a sensitive in vitro fluorescence assay, but whether this was due to TOG1 or the C-terminal linker was not investigated [26]. Recent studies have implicated CLASP family TOG1 domains in non-tubulin binding roles: yeast (*S*.*c*.) Stu1 TOG1 plays a role in kinetochore localization while *H*.*s*. CLASP2 TOG1 regulates CLASP2-dependent effects on microtubule dynamics by inhibiting the activity of *H*.*s*. CLASP2 TOG2 [25,45].

Unlike *H*.*s*. CLASP1 TOG1, the modeling of *H*.*s*. CLASP1 TOG2 and *M*.*m*. CLASP2 TOG3 onto tubulin using the *S*.*c*. Stu2 TOG2-tubulin complex as a guide yielded significant gaps between the second HR triad and αβ-tubulin (Fig 6A). For *H*.*s*. CLASP1 TOG2, the bent architecture of the domain angles HRs D-F away from αβ-tubulin. Interestingly, *H*.*s*.CLASP1 TOG2 has many *S*.*c*. Stu2 TOG2-like tubulin binding determinants across its intra-HR loops including HR A W338 (equivalent to *S*.*c*. Stu2 W341), HR D S457 and V458 (equivalent to *S*.*c*. Stu2 T470 and P471 respectively), HR E R503 (equivalent to *S*.*c*. Stu2 R519), and HR F K534 (equivalent to *S*.*c*. Stu2 R551)(Fig 6B). This suggests that *H*.*s*. CLASP1 TOG2 may engage tubulin across all of its intra-HR loops. If *H*.*s*. CLASP1 TOG2 adheres to a rigid conformation upon tubulin binding, this may in turn drive tubulin into a hyper-curved conformation that exceeds the curve observed in structures solved to date (*e*.*g*. see [36,56]). Recent work has demonstrated that the CLASP family TOG2 domain promotes microtubule rescue and is necessary and sufficient for CLASP-dependent microtubule anti-catastrophe activity [25,26]. The unique bent architecture on the microtubule-binding surface of *H*.*s*. CLASP1 TOG2 and its role limiting catastrophe and promoting rescue may reflect, and be complementary to, the dynamic, curved protofilament architecture observed in both polymerizing and depolymerizing microtubules [55]. The CLASP family TOG3 domain, *H*.*s*. CLASP2 TOG3, has been found to promote microtubule rescue events [25]. Our superpositioning model of *M*.*m*. CLASP2 TOG3 onto tubulin yields an interaction mode distinct from *H*.*s*. CLASP1 TOG2, potentially aligned with its rescue activity (Fig 6A). *M*.*m*. CLASP2 TOG3 HR D is positioned away from β-tubulin and HR F is angled away from α-tubulin. The unique positioning of *M*.*m*. CLASP2 TOG3’s two HR triads relative to one another, and in comparison to the HR triads of *S*.*c*. Stu2 TOG2, leads to a unique positioning of the second HR triad on the surface of α-tubulin. While *M*.*m*. CLASP2 TOG3 has a unique TOG architecture, it retains a set of key intra-HR residues that are positioned equivalent to the tubulin-binding determinants of *S*.*c*. Stu2 TOG2 and are well oriented to engage αβ-tubulin. These include HR A loop W660 (equivalent to *S*.*c*. Stu2 W341), HR B P699 and H700 (equivalent to *S*.*c*. Stu2 A384 and N385 respectively), HR D S781 and L782 (equivalent to *S*.*c*. Stu2 T470 and P471 respectively), and HR E R830 (equivalent to *S*.*c*. Stu2 R519) (Fig 6B).

**Fig 7:**
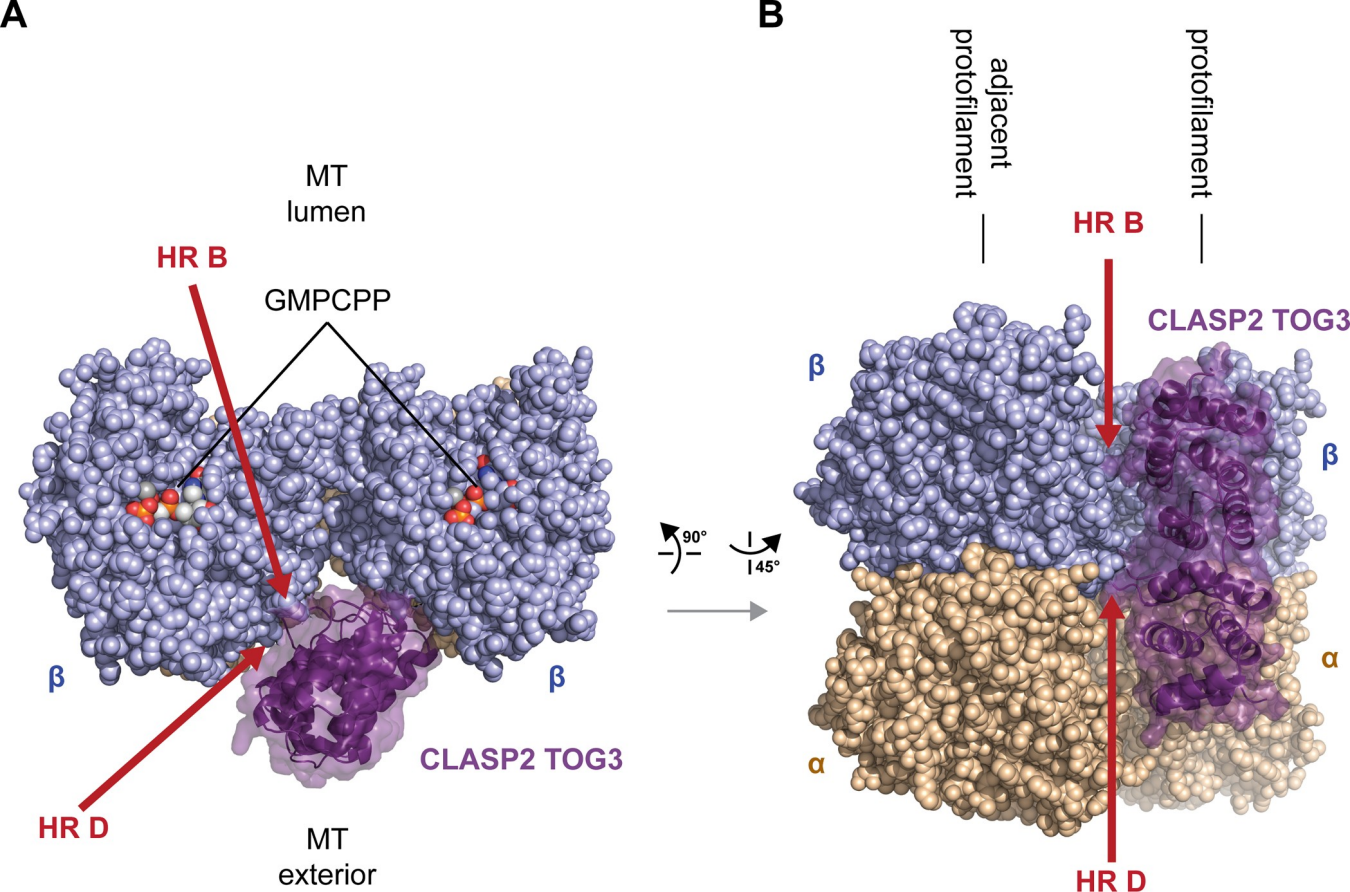
*M*.*m*. CLASP2 TOG3 is predicted to engage laterally associated tubulin on the microtubule lattice. (A) Model of *M*.*m*. CLASP2 TOG3 superpositioned on a microtubule. Shown are two laterally-associated tubulin heterodimers from neighboring protofilaments. *M*.*m*. CLASP2 TOG3 is shown in dark purple, modeled bound to the tubulin heterodimer shown at right (akin to the single-tubulin heterodimer binding mode depicted in Fig 6A). The model generated of *M*.*m*. CLASP2 TOG3 bound to free tubulin (Fig 6A) was superpositioned onto the lattice coordinates of GMPCPP-bound tubulin (PDB accession code 3JAT [53]). The tops of the β-tubulin subunits are shown (plus end oriented towards the viewer), looking into the bore of the microtubule with the luminal region oriented above and the microtubule exterior oriented below. (B) Model as shown in (A), viewed from the microtubule exterior with the plus end oriented up. β-tubulin is shown in lavender, α-tubulin is shown in wheat. Potential *M*.*m*. CLASP2 TOG3 HR B and HR D contacts with the laterally-associated tubulin subunit are demarcated with red arrows.

The distinct architecture of *M*.*m*. CLASP2 TOG3, modeled with a unique lateral shift on the tubulin heterodimer (Fig 6A) suggests that it may be involved in engaging a laterally associated tubulin subunit on the microtubule. To examine this, we used the model generated of *M*.*m*. CLASP2 TOG3 bound to free tubulin (Fig 6A) and superpositioned this onto the lattice coordinates of GMPCPP-bound tubulin (PDB accession code 3JAT [53], see Materials and methods for details). As modeled, *M*.*m*. CLASP2 TOG3 makes contacts with the laterally-associated tubulin subunit on the adjacent protofilament. These contacts involve determinants in *M*.*m*. CLASP2 TOG3 HR B and the unique extended intra-HEAT loop of HR D (Fig 7A and 7B). The potential ability of *M*.*m*. CLASP2 TOG3 to bridge adjacent protofilaments may underlie its ability to promote rescue.

## Conclusion

**Fig 8:**
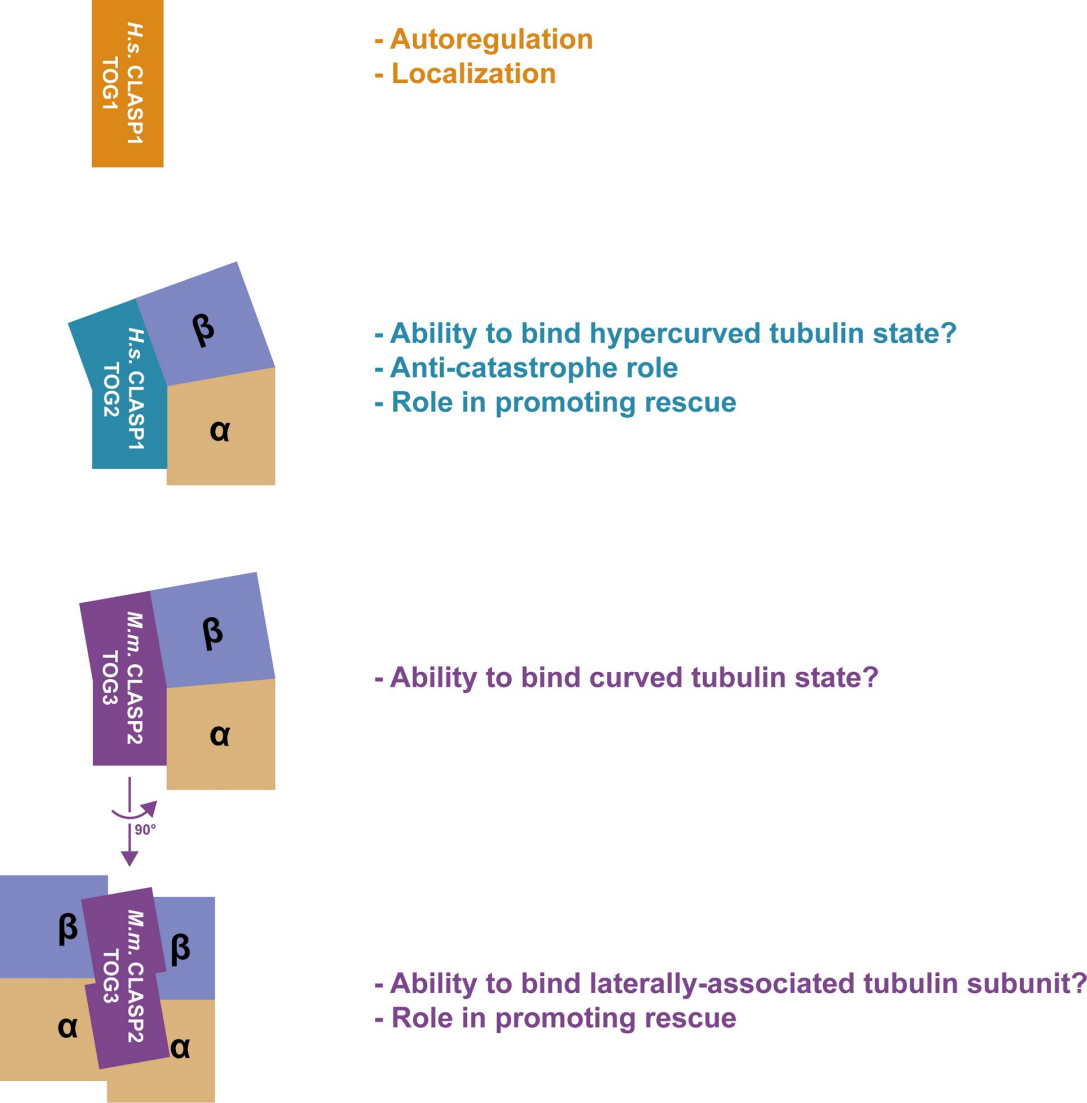
Model of CLASP family TOG domain structures and potential tubulin binding modes. TOG domains and tubulin are colored as presented in Fig 6A. From top to bottom: *H*.*s*. CLASP1 TOG1 (orange) is architecturally similar to the TOG domains of the XMAP215 family member *S*.*c*. Stu2. While no tubulin- or microtubule-binding activity has been detected for CLASP family TOG1 domains, a role in cellular localization and autoregulation has been reported. *H*.*s*. CLASP1 TOG2 (blue) has a convex TOG architecture across its tubulin-binding surface that may be used to engage tubulin in a hypercurved state and may underlie the anti-catastrophe and rescue activity reported for the structurally similar *H*.*s*. CLASP2 TOG2 domain. *M*.*m*. CLASP2 TOG3 (purple) also has a convex architecture across its tubulin-binding surface, but is not bent as dramatically as *H*.*s*. CLASP1 TOG2. The convex *M*.*m*. CLASP2 TOG3 architecture may be used to engage a curved tubulin state. Relative to the other CLASP family TOG domains presented, the *M*.*m*. CLASP2 TOG3 domain has a unique architectural shift in the plane tangential to the microtubule surface and orthogonal to its tubulin-binding surface. This shift may enable it to engage the laterally associated tubulin subunit on the neighboring protofilament and support the reported ability of *H*.*s*. CLASP2 TOG3 to promote microtubule rescue.

We have used crystallography to determine the structures of *H*.*s*. CLASP1 TOG1 and *H*.*s*. CLASP1 TOG2. We find that *H*.*s*. CLASP1 TOG1 and *H*.*s*. CLASP1 TOG2 (presented here) are structurally distinct from one another and from the reported structure of *M*.*m*. CLASP2 TOG3 [44]. However, *H*.*s*. CLASP1 TOG1 is structurally similar to the previously reported structure of *H*.*s*. CLASP2 TOG1 [25] and *H*.*s*. CLASP1 TOG2 is structurally similar to the previously reported structure of *H*.*s*. CLASP2 TOG2 [44] as well as the structure of *H*.*s*. CLASP1 TOG2 determined in a different space group [34]. This suggests that animal CLASP family TOG domains are found arrayed in a specific, conserved, structural order across paralogs and species. While *H*.*s*. CLASP1 TOG1 has a canonical TOG structure similar to the tubulin-binding TOG domains of the XMAP215 family member *S*.*c*. Stu2 [36,37], *H*.*s*. CLASP1 TOG1 has distinct determinants across the intra-HR loops that likely explain why no tubulin or microtubule-binding activity has been detected for animal CLASP family TOG1 domains [25,41,44]. In contrast, *H*.*s*. CLASP1 TOG2 is curved across the tubulin-binding surface and our structural modeling suggests that it may engage tubulin in a hyper-curved state. The previously reported structure of *M*.*m*. CLASP2 TOG3 also reveals a bent architecture [44], both along its tubulin-binding intra-HR loops surface, as well as along an orthogonal plane that, in our analyses, potentially positions the domain to engage laterally associated tubulin on an adjacent protofilament. Our comparison of these structures to other CLASP family TOG domain structures and to XMAP215 family tubulin-binding TOG domains has highlighted a number of features of the CLASP family TOG array: 1) each TOG domain along the animal CLASP family TOG array has a unique architecture (our work and [25,34,41,44]), 2) the structure of each specific TOG domain along the animal CLASP family TOG array is well conserved across species, across paralogs, and across different crystal space groups (our work and [25,34,41,44]), 3) the unique structures of these TOG domains and their respective conserved determinants (our work and [25,34,41,44]) correlates with each TOG domain having unique activities: CLASP family TOG1 domains have been reported to have non-tubulin binding roles in kinetochore localization and relieving CLASP from an auto-inhibited state [25,45], the CLASP family TOG2 domain plays a role in limiting catastrophe and stimulating rescue [25,26], and the CLASP family TOG3 domain plays a role in promoting rescue [25], 4) the unique structures of the animal CLASP family TOG2 and TOG3 domains (our work and [34,44]) predict distinct modes of tubulin binding on the microtubule lattice: the CLASP family TOG2 domain may preferentially engage a hyper-curved tubulin state and the CLASP family TOG3 domain may bridge adjacent tubulin subunits on neighboring protofilaments (Fig 8). It is of note that our work analyzes the structure of individual CLASP family TOG domains and does not examine potential synergy between TOG domains or with the linkers that bridge them. Prior work has demonstrated that XMAP215 family TOG arrays cannot tolerate permutations in the array, specifically those that alter the order of, or exclude structurally distinct TOG domains [38]. Similarly, the liker spacing between TOG domains has been found to be important, likely reflecting distance constraints imposed as TOG-bound tubulins form tubulin-tubulin interactions at polymerizing microtubule ends [37,57]. However, it is of note that intrinsic microtubule regulatory activities have been ascribed to individual *H*.*s*. CLASP2 TOG constructs that can track microtubule plus ends via the inclusion of a EB-binding motif, (CLASP2 TOG2: ability to suppress catastrophe and stimulate rescue; CLASP2 TOG3: ability to enhance rescue) [25].

While our work highlights distinct structural features of the animal CLASP family TOG array, a number of key questions remain outstanding. 1) What factor does the CLASP family TOG1 domain bind? No tubulin-binding activity has been ascribed to the animal CLASP family TOG1 domain. Instead of binding tubulin, the animal CLASP family TOG1 domain’s conserved intra-HR loops may be involved in binding a kinetochore factor [45,58], relieving the auto-inhibition of the CLASP family TOG2 domain’s activity [25], binding actin [59], or a yet to be determined factor. 2) What is the structural conformation of a CLASP family TOG2- and a TOG3-tubulin complex as found on a microtubule? Will these structures yield insight into novel structural states of the tubulin heterodimer and will these states inform the mechanisms by which the animal CLASP family TOG2 and TOG3 domains affect microtubule dynamics? 3) While the CLASP family TOG array is composed of structurally distinct TOG structures arranged in a specific order, can the array function properly if shuffled or do the unique architectures play synergistic order- and spatial-specific roles along the polarized microtubule lattice? 4) While the structural nature of the animal CLASP family’s three TOG domains (TOGs 1–3) has been elucidated, little is known about its C-terminal CLIP-ID [16]. The CLIP-ID is predicted to be composed of HRs and analysis suggests that it may conform to a TOG architecture as found in the fifth TOG domain of XMAP215 family members that have a pentameric TOG array [38]. Whether this is the case, whether the CLIP-ID has tubulin- or microtubule-binding activity, and how CLIP-170 binds the CLIP-ID remains to be determined.

Our work highlights the structural diversity of the TOG domains that comprise the CLASP family TOG array. This adds to our growing understanding of TOG domain structural diversity and the specific role these structures play when arrayed in different regulators of microtubule dynamics, including the CLASP family, the XMAP215 family of microtubule polymerases, and the Crescerin family that regulates microtubule structure and dynamics in cilia [60–62].
